# Supplementary material for: Integrating Metabolomics and Gut Microbiota to Identify Key Biomarkers and Regulatory Pathways Underlying Metabolic Heterogeneity in Childhood Obesity
Source: Nutrients. 2025 May 30;17(11):1876. doi: 10.3390/nu17111876 (PMC12158115; doi:10.3390/nu17111876)
Supplement: Supplementary file 1 [file nutrients-17-01876-s001.zip › nutrients-3615741-supplementary-1.pdf]

Spearman correlation analysis was performed on all the differential metabolites (VIP>1, FDR<0.05, FC>1.2) of the OVOB/NOR group analyzed by OPLS-DA in this study and the differential bacterial genera between the two groups analyzed by Maaslin2. The results showed that a total of 21 differential metabolites were associated with the differential bacterial genera. Please refer to **Figure S1.** for details.

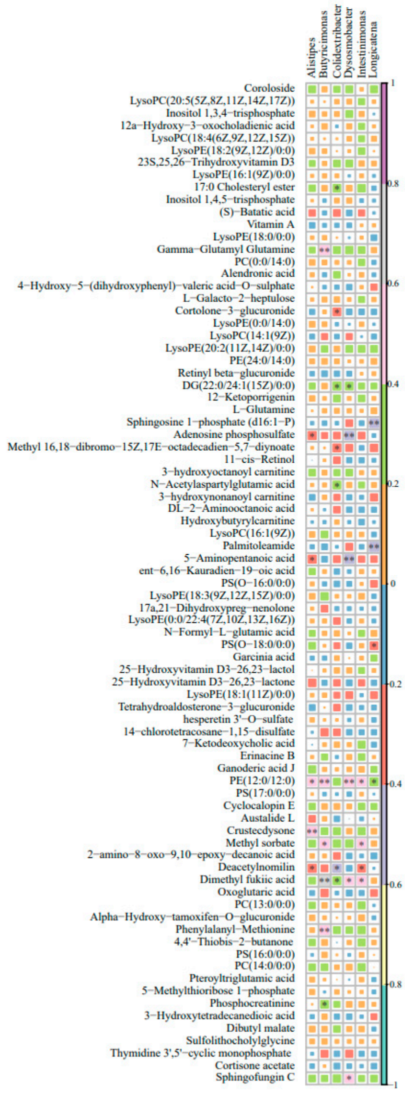

Figure S1. Spearman association between all different metabolites and different bacteria in OVOB/NOR group. Square size represents the absolute value of the correlation coefficient (larger size = stronger association). *indicates that the correlation is statistically significant ($p < 0.05$), **indicates that the correlation is statistically significant ($p < 0.01$).

Model validity was rigorously confirmed using permutation testing (200 iterations, $p < 0.001$), demonstrating resistance to overfitting (Figure S2). These statistically robust OPLS-DA models establish a credible foundation for identifying biologically relevant differential metabolites between comparison groups.

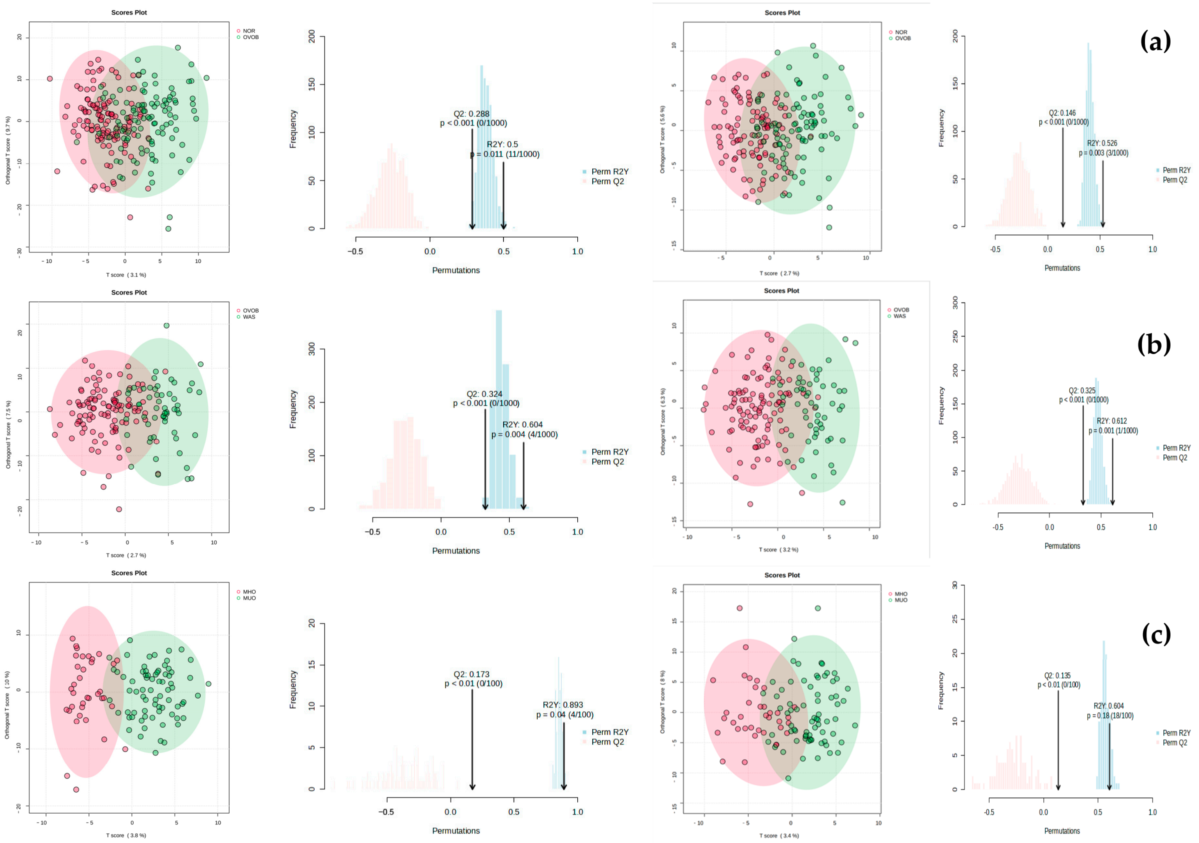

Figure S2. OPLS-DA model and permutation testing results of samples from comparison groups. Columns 1 and 3 are the results of metabolite group difference analysis using OPLS-DA under positive and negative ion modes, respectively. Columns 2 and 4 are the results of model permutation testing under positive and negative ion modes, respectively. (a) Description of the results of the OPLS-DA and permutation tests for the OVOB/NOR comparison group; (b) description of the results of the OPLS-DA and permutation tests for the OVOB/WAS comparison group; (c) description of the results of the OPLS-DA and permutation tests for the MUO/MHO comparison group.
